# Solvation free energies and partition coefficients with the coarse-grained and hybrid all-atom/coarse-grained MARTINI models

**DOI:** 10.1007/s10822-017-0059-9

**Published:** 2017-09-05

**Authors:** Samuel Genheden

**Affiliations:** Department of Chemistry and Molecular Biology, University of Gothenburg, Box 462, 405 30 Gothenburg, Sweden

**Keywords:** Solvation free energies, Partition coefficients, Coarse graining, MARTINI model, Hybrid modeling

## Abstract

We present the estimation of solvation free energies of small solutes in water, *n*-octanol and hexane using molecular dynamics simulations with two MARTINI models at different resolutions, viz. the coarse-grained (CG) and the hybrid all-atom/coarse-grained (AA/CG) models. From these estimates, we also calculate the water/hexane and water/octanol partition coefficients. More than 150 small, organic molecules were selected from the Minnesota solvation database and parameterized in a semi-automatic fashion. Using either the CG or hybrid AA/CG models, we find considerable deviations between the estimated and experimental solvation free energies in all solvents with mean absolute deviations larger than 10 kJ/mol, although the correlation coefficient is between 0.55 and 0.75 and significant. There is also no difference between the results when using the non-polarizable and polarizable water model, although we identify some improvements when using the polarizable model with the AA/CG solutes. In contrast to the estimated solvation energies, the estimated partition coefficients are generally excellent with both the CG and hybrid AA/CG models, giving mean absolute deviations between 0.67 and 0.90 log units and correlation coefficients larger than 0.85. We analyze the error distribution further and suggest avenues for improvements.

**Electronic supplementary material:**

The online version of this article (doi:10.1007/s10822-017-0059-9) contains supplementary material, which is available to authorized users.

## Introduction

Molecular simulations are nowadays readily used as complements to wet-lab experiments and can be used to understand atomistic-level interactions as well as to calculate thermodynamic and kinetic quantities. Applications particularly relevant for the current study are the estimation of pharmacokinetics of novel drug candidates and the accumulation of small molecules in the biota [1, 2]. The basis of molecular simulations is a physical model of the system of interested that is propagated with either molecular dynamics (MD) or Monte Carlo methods [3]. The model is typically a molecular mechanics force field that allows the system to be simulated at sufficient detail and simultaneously allowing the simulations to reach appropriate time scales. However, there is still a hierarchy of different models to choose from within the molecular mechanics framework. The most detailed models are the all-atom (AA) models that treat each atom individually, thus providing a high degree of accuracy in theory but with the caveat that these models become too costly for large systems at long time scales. To circumvent this caveat, coarse-grained (CG) models have been developed that group atoms into pseudo-particles or beads [4, 5]. This drastically reduces the number of particles that needs to be propagated and at the same time smoothens the energy landscape and thereby further speeding up the simulations [6]. Although CG models have been used in numerous applications, they are inherently less accurate than AA models. It is for instance impossible to distinguish between similar small compounds [7] and problems with retaining a proper protein structure have been reported [8], which can be solved by applying an elastic network model [9]. Therefore, hybrid AA/CG models have been suggested that combines an AA model of the most important molecule(s) with a CG model for the majority of the molecules (the solvent molecules) [10–15]. Such models retain the accuracy of the AA model for the most important part but potentially enjoy the speed of the CG model.

**Fig. 1:**
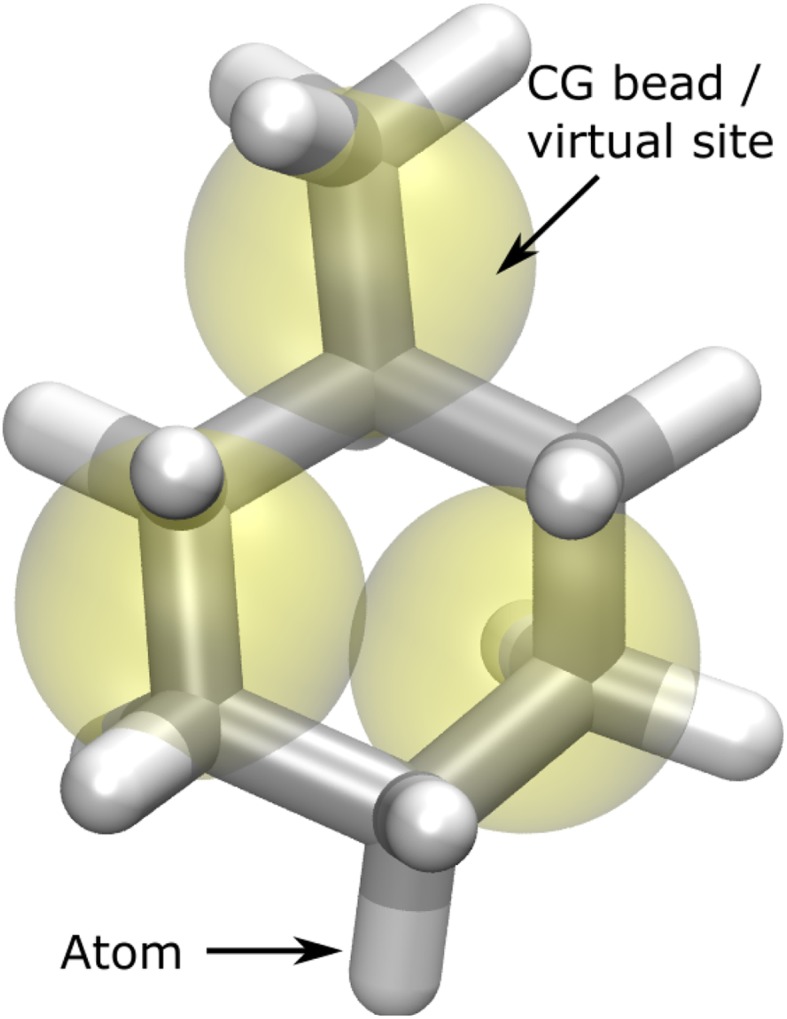
The MARTINI AA/CG model of methylcyclohexane. The AA model is shown as sticks and the CG model is shown as yellow semi-transparent balls. In the simulations, the CG beads are represented as virtual sites (VS) that are mapped on a set of atoms

The MARTINI model is one of the most widely used CG models and has parameters for e.g., proteins, lipids, sugars and nucleic acids [6, 16]. In addition, a program to automatically parameterize small, organics molecules was recently presented [7]. The CG model is based on a 4:1 mapping, i.e. on average four heavy atoms are mapped to a single CG bead, except for ring structures were only two heavy atoms are mapped to a CG bead [6]. A hybrid AA/CG model has been presented [12, 17, 18] and extended to an adaptable AA/CG border [19], but the model has not been extensively used or tested. The AA/CG model of a solute combines the atoms from the AA model with CG beads that are represented as virtual sites, mapped on top of the atoms (see Fig. 1) [12]. A recent large-scale test of solvation free energies in octanol and water found considerable errors in the MARTINI CG estimates [7], and a hybrid AA/CG model could be a solution to these deficiencies. Alternatives to the MARTINI AA/CG model exist such as the GROMOS and ELBA models [13, 15]. The latter model has for instance been used to simulate proteins and membrane permeability [20]. In contrast to the MARTINI AA/CG model, the ELBA AA/CG model has been extensively benchmarked on solvation free energies and partition coefficients, showing promising results [21, 22].

In this paper, we perform large-scale benchmark calculations of solvation free energies and partition coefficients using the MARTINI model, which is a common approach to validate a force field [23–25]. We investigate if the estimates of the CG model can be improved by using a hybrid AA/CG model of the solutes. Furthermore, we compare estimates with two different MARTINI water models, one non-polarizable and one polarizable. Finally, we also compare our results to earlier benchmarks with the MARTINI CG and ELBA AA/CG models [7, 21].

## Methods

### Models

Solute molecules were selected from the Minnesota solvation database (version 2012) [26], containing experimentally determined solvation free energies in a range of solvents. We selected solutes that had a determined solvation free energy in water, octanol or hexane. Furthermore, we limited the set of solutes to those that had at most ten heavy atoms and excluded hydrogen, water and tetramethylsilane in order to make the set identical to the benchmark set used to evaluate the ELBA AA/CG model [21]. In addition, we had to exclude methane, naphthalene, trimethylphosphate due to problems of generating automatic coarse-grained (CG) models and acetophenone, nitrobenzene, 2-methyl-1-nitrobenzene, methyl benzoate and 2,6-dichlorobenzonitrile due to instabilities with the automatic CG models. This leaves the total number of unique molecules to 160.

In order to setup the models and calculations for this amount of solutes, a semi-automatic procedure was implemented:

1. SMILES strings [27] were retrieved from the chemical names using the ChemSpider server [28]. For a few solutes this failed and the SMILES had to be manually corrected.
2. Using the SMILES string as input, a CG model of the solute was created with the auto_martini program [7]. This will also create a coordinate file for the all-atom (AA) model and a mapping from CG to AA.
3. Using the AA coordinates as input, an AA model of the solute is created with the antechamber and parmcheck programs [29]. The AA model is thus described by the general Amber force field (GAFF) with AM1-BCC charges [30, 31].
4. Combining the AA and CG models to a hybrid AA/CG model using the parmed libraries [32]. A MARTINI AA/CG model consists of the AA model plus the CG model represented as virtual sites (VS) [12]. The VS sites are mapped on a set of heavy atoms and only interact with solvent beads, not with each other. This is illustrated in Fig. 1.
5. Solvating either the CG or AA/CG solutes using pre-equilibrated boxes with CG solvent molecules. The length of the box for the solvated system was 3.5 nm.

To summarize the hybrid model: the solute is modeled at both a CG and atomistic level, with the CG beads represented as virtual sites (see Fig. 1). All solvent molecules are CG. The scripts to setup the simulations are publicly available from Github (http://www.github.com/sgenheden).

We used two CG models of water: the standard, non-polarizable MARTINI water model and the polarizable PW model [33]. The non-polarizable model consists of a single bead, representing four real water molecules. The bead is uncharged and is of the P4 atom type [6]. The PW model also represents four real water molecules but consists of three sites: the central site is uncharged and is of the special POL atom type. The two sites bonded to this are charged but do not interact through a Lennard–Jones potential. The polarizability of the model comes from the angle potential connecting the beads and the constrained bond lengths [33]. The MARTINI octanol model represents one real octanol molecule and consists of two uncharged beads, one polar (P1 type) and one apolar (C1 type). A MARTINI hexane model is publicly unavailable, so for this solvent we used a model consisting of two uncharged and apolar beads (C1 type), which represents one real hexane molecule. This model has a liquid density of 610.8 g/L compared to the experimental density of 660.6 g/L [34], and an enthalpy of vaporization of 33.7 kJ/mol compared to an experimental enthalpy of 31.6 kJ/mol [35], thereby showing its suitability.

In the CG simulations, the standard MARTINI non-bonded functional form (see Table 1) was used. However, in the AA/CG simulations, tabulated potentials were used to allow different non-bonded functional forms for the different types of pair interactions [12, 17]. For the AA/CG simulations with the polarizable water model, we used a suggested electrostatic coupling scheme with an internal dielectric constant of 1.45 [17]. In all cases a 1.2 nm cut-off was used. The functional forms used are summarized in Table 1.

**Table 1:** Summary of non-bonded potentials used in the simulations

Pair type	CG simulations	Polarizable CG simulations	AA/CG simulations	Polarizable AA/CG simulations
AA–AA	–	–	Cut–off (ε = 1.0)	Cut–off (ε = 1.0)
AA–CG	–	–	Zero	RF (ε = 1.45)/Shifted LJ
AA–VS	–	–	Zero	Zero
CG–CG	RF (ε = 15)/Shifted LJ	RF (ε = 2.5)/Shifted LJ	RF (ε = 15)/Shifted LJ	RF (ε = 2.5)/Shifted LJ
CG–VS	–	–	RF (ε = 15)/Shifted LJ	Zero/Shifted LJ
VS–VS	–	–	Zero	Zero

First the electrostatic function is given and then, if it is different from the former, the van der Waals function

RF = reaction field electrostatics with solvent dielectric equal to infinity. Shifted LJ = shifted Lennard–Jones from 0.9 to 1.2 nm. Cut-off = plain cut-offs. Zero = interaction is zero everywhere. ε is the internal dielectric constant

### Simulations

The solvated systems were minimized with 1000 steps of steepest descent, followed by 1.5 ns equilibration in the NPT ensemble. The timestep was 20 and 2 fs in the CG and AA/CG simulations, respectively. For some solutes, the timestep had to be decreased to 1 fs in the AA/CG simulations due to system instabilities. The temperature and pressure were controlled with weak-coupling algorithms [36]. The relaxation time was 1 and 12 ps for the thermostat and barostat, respectively. Two independent equilibrated systems for each solute were created in this way by translating the solute in the box prior to solvation and by assigning different initial velocities to the particles.

The equilibrated systems were subjected to free energy simulations, in which the solute was step-wise decoupled from the environment, by introducing a coupling parameter that scales the interaction between the solute and the environment [37]. This was accomplished with 21 windows, evenly distributed from 0 to 1, the electrostatics and van der Waals interactions were decoupled simultaneously and softcore potentials [38, 39] were used. At each value of the coupling parameter, the system was simulated for 2.5 ns in the NPT ensemble. The initial 500 ps were considered to be equilibration and the sampling frequency was 5 ps. The settings were otherwise identical to the equilibration simulation. From these simulations the solvation free energies, Δ*G*_solv_, were estimated with the Bennet acceptance ratio (BAR) method [40] as the negative of the decoupling free energy.

### Error analysis

The agreement between the experiments and the computational estimates were quantified by calculating the mean absolute deviation (MAD), Pearson’s correlation coefficient (*R*), Kendall’s τ and the slope of the correlation curve.

The BEDROC (Boltzmann-enhanced discrimination of receiver-operating characteristic) metric was computed for different chemical groups as outlined previously [41, 42]. The Checkmol program [43] (version 0.5) was used to classify the compounds and the BEDROC values were computed with the CROC python package [44] (version 1.1). The uncertainties of the BEDROC values were estimated using 500 iterations of bootstrapping.

## Results and discussion

### CG models

We used a recently published program [7] to produce the CG models necessary for the solvation free energy calculations. As described in the previous section, there are 160 unique solutes and these are mapped to only 40 unique CG models. A majority of the solutes (103) are mapped to a single bead, which of course is an effect of the limit on the molecular size of the solutes in the test set. In fact, 16 solutes were mapped to the C5 type, a non-polar bead. Furthermore 49 solutes are mapped to two beads and eight solutes to three beads. These numbers already highlight a drawback of the CG approach, and a potential use of a hybrid AA/CG approach. However, the necessity to create a coarse-grained (CG) model for each solute is potentially a drawback of the MARTINI hybrid model, because the quality of the CG model of the solute will affect the simulations. This is in contrast to for instance the GROMOS and ELBA hybrid models, where the coupling between the AA and CG parts is direct and there is no need to make a CG model.

### Solvation free energies

**Fig. 2:**
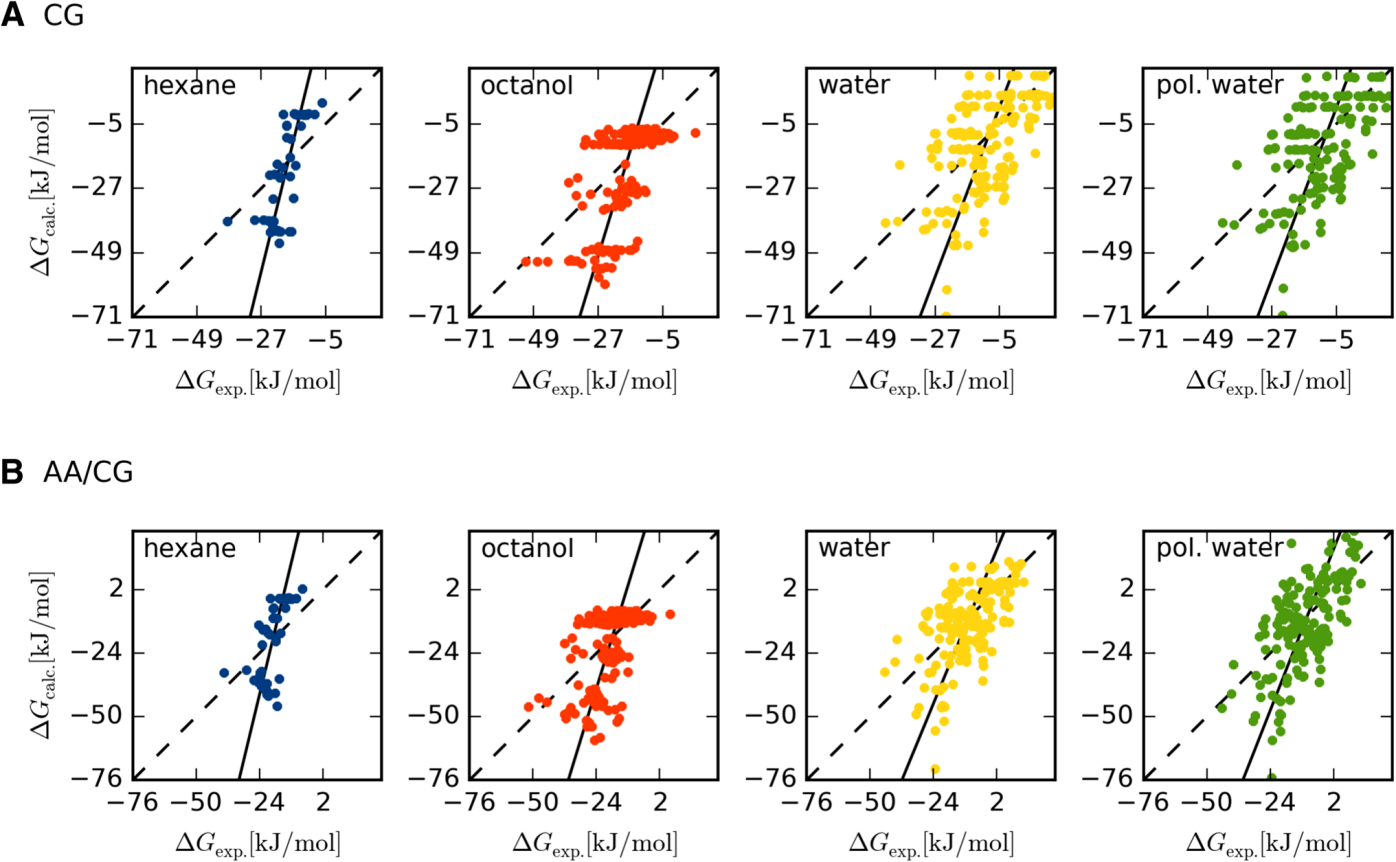
Correlation between experimental and calculated solvation free energies for **a** CG solutes and **b** AA/CG solutes. The solvent is indicated in the *upper-left corner*. The *dashed lines* indicate a perfect correlation line and the *filled lines* indicate the observed correlation

The calculated solvation free energies (Δ*G*_solv_) are plotted against experiments in Fig. 2 and quality metrics are listed in Table 2. Full results can be found in the supplementary material. In Table 2, we have also listed the number of solute molecules that have experimental data in the different solvents, which ranges from 51 for hexane to 160 for water. It should be noted that we computed the solvation free energies in anhydrous octanol, a common and well-proven approximation in the literature [7, 42, 45].

**Table 2:** Statistics of the performance for the solvation free energy (Δ*G*_solv_) and partition coefficient (log *P*) calculations

	N solutes	Δ*G*_solv_				log *P*			
		MAD [kJ/mol]	R	τ	Slope	MAD	R	Accuracy^a^ (%)	Slope
CG solutes									
Hexane	51	11.8	0.75	0.54	0.06	0.87	0.87	84	0.61
Octanol	158	11.6	0.59	0.46	0.09	0.67	0.86	92	0.44
Water	160	11.4	0.55	0.36	0.14				
Pol. water	160	11.6	0.55	0.36	0.14				
AA/CG solutes									
Hexane	51	11.1	0.67	0.47	0.06	0.90	0.87	86	0.57
Octanol	158	10.7	0.55	0.43	0.09	0.71	0.86	92	0.40
Water	160	10.4	0.56	0.38	0.17				
Pol. water	160	11.4	0.64	0.42	0.15				

^a^Defined as the percentage of estimate with the correct sign

Starting with the pure coarse-grained (CG) simulations for hexane, there is a considerable difference between the calculations and experiments as shown by the mean absolute deviation (MAD) of 12 kJ/mol. However, the relative performance is rather good: the correlation coefficient (*R*) is 0.75 and significant, and Kendall’s τ is 0.54, indicating that most of the calculated Δ*G*_solv_ of the solutes are correctly ordered. Still, as can be seen from the correlation plot in Fig. 2, the slope is close to zero (0.06). This partly stems from the fact that many of the solutes are mapped to the same CG representation as discussed above. This is shown as the horizontal smears in the correlation plot, i.e. although the experimental Δ*G*_solv_ is different the calculated Δ*G*_solv_ is identical for a range of solutes. Thus there seems to be no systematic error that can be fixed by for instance scaling the solute–solvent interactions, which has been a successful strategy to improve hybrid models [14, 21].

The situation is similar for CG estimates in octanol: the MAD of 12 kJ/mol indicates a considerable difference between calculations and experiments, but the *R* of 0.59 and τ of 0.46 indicates a decent correlation and ranking. However, the horizontal smear is more pronounced in the correlation plot in Fig. 2, due to the larger number of solutes for this solvent and the slope is again close to zero (0.09). For the CG solutes in water the estimated Δ*G*_solv_ is more evenly spread around the ideal correlation line, although the MAD is 11–12 kJ/mol and the τ of 0.36 is considerably worse than for hexane and octanol. For completeness, we also computed hydration free energies with a polarizable water model. However, this model was developed to deal with bad screening in the water phase, and hence we do not expect any difference at all between the two water models because the benchmark set consists of neutral solutes. Correctly, we also observe a MAD between Δ*G*_solv_ calculated with the non-polarizable model compared to the polarizable to be only 0.6 kJ/mol, and probably not statistically significant. Therefore, for small and neutral organic molecules, there is no point of using the slightly more expensive polarizable model.

The results discussed above agree well with a recent benchmark of computed solvation free energies in water and octanol [7]. For 354 compounds in water, *R* was 0.56 and MAD 11.2 kJ/mol and for 69 compounds in octanol, the *R* was 0.51 and MAD was 12.1 kJ/mol. The error was partly attributed to the limited fluidity range of the Lennard–Jones potential that is the basis of the MARTINI model, and partly to an apparent non-additivity of the solvation free energy for individual beads [7]. It was also pointed out in one of the earliest MARTINI publications that the model performed badly when predicting solvation free energies [6].

As a potential remedy to the deficiency of the pure CG model, we sought to estimate the solvation free energies with the AA/CG hybrid model. Furthermore, an AA/CG hybrid model has the potential to be particularly useful as all the solutes will be technically distinguishable. However, as can be seen from the results in Table 2 and Fig. 2, there is very little difference between the estimates with the CG and the AA/CG models. The AA/CG models result in MAD that ranges between 10 and 12 kJ/mol, *R* between 0.55 and 0.67, and τ between 0.38 and 0.47, in the different solvents. This is very similar to the results with the CG models and the small individual differences for the different solvents are probably not statistically significant. The individual differences between the estimates with CG and AA/CG solutes are further quantified in Table 3. The MAD over all the solutes ranges between 2 and 4 kJ/mol, with a small systematic component as seen from the mean signed deviation (MSD) of −1 to −3 kJ/mol (indicating that the hybrid AA/CG estimates are more positive than the CG estimates). The correlation is very strong between the two sets of estimates with *R* > 0.95 for all solvents. The only clear and interesting difference is seen for the estimates with the polarizable water model. Here, we have chosen the electrostatic coupling suggested in a previous study [17] where the charged beads of the CG water are directly interacting with the charges on the atoms of the solute, while the van der Waals interaction is still between the CG solvent beads and the virtual sites of the solutes. This leads to a direct coupling between the CG and AA levels and as can be seen in Fig. 2b leads to a decrease in the horizontal smear in the correlation plot, i.e. similar solutes are truly distinguishable. In the other solvents, the interaction between the AA and CG levels is indirect through the virtual sites and hence basically on the CG level. The CG–VS interactions are also the ones being decoupled in the free energy simulations and therefore, it is not entirely surprising that the CG and AA/CG estimates are very similar. Thus it seems that a polarizable solvent, or at least one with some electrostatics is preferable when coupling to an AA/CG hybrid solute model. This also illustrate the usefulness of the polarizable water model as compared to the pure CG simulations, where there was virtually no difference between the two water models (as expected).

**Table 3:** Comparison between the CG and AA/CG estimates in the different solvents

	MAD [kJ/mol]	MSD [kJ/mol]	*R*
Hexane	3.0	−2.6	0.98
Octanol	2.2	−1.6	0.98
Water	3.3	−1.7	0.96
Pol. water	4.2	−0.9	0.95

The hybrid AA/CG results are significantly worse than what was achieved with the hybrid ELBA model [21]. For 168 solutes in water, the MAD in Δ*G*_solv_ was 4 kJ/mol and *R* 0.93. Similar results were obtained with 166 solutes in octanol and 54 solutes in hexane; the MAD was 4 and 3 kJ/mol for octanol and hexane, respectively, with *R* values 0.91 and 0.78. The ELBA AA/CG model couples the interaction (both van der Waals and electrostatics) between the CG solvents and the AA solute directly through standard potentials [15], which again indicates that a direct AA–CG interaction is preferable.

### Partition coefficients

**Fig. 3:**
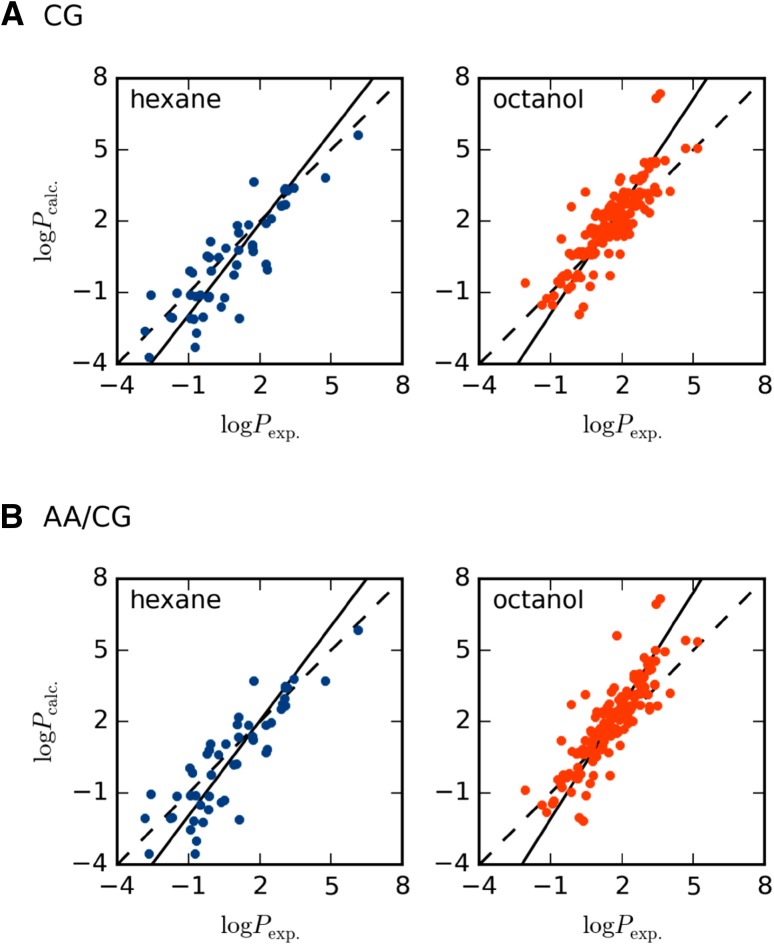
Correlation between experimental and calculated partition coefficients for **a** CG solutes and **b** AA/CG solutes. The partition coefficient is between water and the solvent indicated in the *upper-left corner*. The *dashed lines* indicate a perfect correlation line and the *filled lines* indicate the observed correlation

Using the solvation free energies in water and octanol or hexane, we can form partition coefficients as in

$$\log P=\frac{{\Delta {G_{\text{solv}}}({\text{water}}) - \Delta {G_{{\text{solv}}}}({\text{S}})}}{{2.3RT}}$$

where S is either octanol or hexane, *R* is the gas constant and *T* the absolute temperature. Here we have chosen to use the hydration free energies estimated with the non-polarizable water model, as this water model has a similar theoretical underpinning as the octanol and hexane models. In fact, using the polarizable water model does not affect the results much (not shown) as expected from the very similar free energy estimates with the two models. The correlation plots are shown in Fig. 3 and quality metrics are listed in Table 2.

In contrast to the solvation free energies, the computed partition coefficients agree well with experiments. For the CG solutes, the MAD is less than 0.9 log units and the correlation is 0.87 and 0.86 for water/hexane and water/octanol, respectively. The accuracy, here defined as the percentage of solutes with correctly predicted sign is 84 and 92% for the water/hexane and water/octanol partition coefficients, respectively, indicating that most of the estimated coefficients have the correct sign. Finally, as can be seen from the correlation plot in Fig. 3, the slope is considerably closer to one for both solvents, when comparing to the solvation free energies. These results are not entirely surprising, and are in fact a re-confirmation of the automatic parameterization procedure that is based on a rough estimate of the water/octanol partition coefficients. The same report that presented the parameterization procedure showed an *R* of 0.91, a MAD of 0.57 log units and an accuracy of 98% for the estimation of water/octanol partition coefficients for 653 neutral compounds [7]. Still, this shows that there is a considerable cancellation of errors in the calculations: it appears that we might obtain correct answers for the wrong reasons. The far from unity slope of the solvation free energies shows that there is some physics in the MARTINI model that is not captured correctly but this error is almost entirely cancelled when taking the difference between two solvents.

Given that the solvation free energies were so similar when comparing the CG and AA/CG solutes, it is natural that also the partition coefficients are very similar. The MADs are slightly higher for the AA/CG solutes, but the differences are probably not significant. The *R* and the accuracy are also very similar. Again, this shows that the majority of the physics is determined by the interaction between the CG solvent beads and the VS on the solutes, and that the underlying AA model affects the results very little. The performance of the hybrid model is similar to the hybrid ELBA model that gave a MAD of 0.86 and 0.66 log units for water/octanol and water/hexane partitioning, respectively, and an accuracy of 92 and 80% [21].

### Error analysis

In order to obtain a deeper understanding of the errors, we performed a BEDROC analysis on the hybrid AA/CG predictions of the solvation free energies in octanol and water. The number of solutes with data for hexane was too low to make a detailed analysis. Based on a grouping of the error distribution, a BEDROC analysis determines if a group performed worse than the other groups [41]. Here, we divided the compounds into groups based on what chemical groups they contain or what chemical classes they belong to. For the compounds with experimental solvation free energies in either octanol or water, we identified 16 such groups that contained at least five compounds, as seen in Table 4. The largest group is the aromatic compounds with 36 compounds whereas the groups of aldehydes, carboxylic acids and nitro compounds only contain five compounds.

**Table 4:** BEDROC analysis and mean signed deviation (MSD) in kJ/mol for identified chemical groups

	N	Uniform^a^	Octanol		Water		Pol. water	
			BEDROC	MSD	BEDROC	MSD	BEDROC	MSD
Alcohol	16	0.43	0.53	9.1	**0.62**	10.9	0.44	7.8
Aldehyde	5	0.42	0.39	1.8	0.45	7.4	0.22	−3.1
Alkane	17	0.44	0.17	0.5	0.24	0.5	0.36	5.6
Alkene	10	0.43	0.26	−2.8	0.32	−3.1	0.35	−0.9
Alkyl bromide	10	0.43	0.32	−3.0	0.28	−1.5	0.32	−1.4
Alkyl chloride	6	0.42	0.18	−0.6	0.25	0.3	0.27	−0.6
Amine	12	0.43	0.51	0.6	0.44	0.6	0.53	2.7
Aromatic compound	36	0.46	**0.85**	−18.9	**0.66**	−14.0	**0.69**	−17.4
Carboxylic acid	5	0.42	**0.66**	15.3	**0.73**	16.4	0.50	9.5
Carboxylic acid ester	8/10^b^	0.43	0.19	−2.7	0.28	−4.4	0.40	−9.7
Ether	15	0.43	0.44	−4.1	0.41	−2.5	0.49	−1.8
Halogen derivative	16	0.43	0.41	−2.4	0.46	−1.3	0.41	−3.2
Heterocyclic compound	14	0.43	**0.78**	−17.7	**0.83**	−18.9	**0.85**	−21.4
Ketone	9	0.43	0.31	2.6	0.37	5.5	0.32	0.9
Nitro compound	5	0.42	0.25	2.9	0.10	−1.1	0.14	−2.5
Phenol	7	0.43	0.39	−5.7	0.34	−0.3	0.28	−7.5

The observed BEDROC value is shown and the values that are significantly larger than the value from an analytical, uniform distribution are marked in bold

^a^The uniform, analytical BEDROC value

^b^Group of carboxylic acid esters contain eight compounds in octanol and ten in water

For each of these groups, we computed the BEDROC value (listed in Table 4) and compared it to an analytical estimate that assumes a uniform predictive performance for all groups. For the predictions of Δ*G*_solv_ in octanol, we observe a BEDROC value larger than the analytical value for aromatic compounds, carboxylic acids and heterocyclic compounds. These groups also show a large systematic deviation as shown by the MSD between 15 and 19 kJ/mol. Interestingly, the systematic deviation for carboxylic acids is positive, indicating that the estimates are too positive compared to the experimental data, whereas the aromatic and heterocyclic compounds show a negative MSD, indicating that those estimates are too negative compared to the experimental data. Similar trends are seen for the non-polarizable water, with the addition that also the alcohols have an observed BEDROC that is significantly larger than the analytical value. The alcohols display, similarly to the carboxylic acids, a positive MSD (10.9 kJ/mol). Encouragingly, the MSD is much smaller for alcohols and carboxylic acids when we analyze the errors made with the polarizable water model. Furthermore, these groups do not have an observed BEDROC value that is significantly larger than the analytical value. This indicates that estimates of some highly polar compounds are improved when using a direct coupling between the AA and CG levels. Still, the aromatic and heterocyclic compounds show large MSD and have a high BEDROC value.

Thus this analysis reveals that ring compounds are particularly difficult to estimate with the AA/CG model (and with the CG model as well, not shown). We can arrive at a similar conclusion by repeating the BEDROC analysis and instead grouping the compounds by which beads they consist of (see supplementary material). For compounds containing the SNa and SN0 beads (intermediate polar beads used in ring compounds) the MSD is lower than −20 kJ/mol for both octanol and the two water models. These poor estimates are shown in Fig. 2 as the estimates below the correlation line in the lower-right corner. Such poor estimates were also seen in the correlation plots in the previous benchmark study and were mainly attributed to the non-additivity of the solvation model for individual beads [7]. Here, we identify a re-parameterization of ring compounds as a potential avenue to improve the MARTINI CG model.

## Conclusions

In this report we presented estimates of solvation free energies in hexane, octanol and water along with estimated water/hexane and water/octanol partition coefficients. These estimates were produced with free energy simulations employing a MARTINI CG or MARTINI AA/CG model of the solutes and a CG representation of the solvent molecules. Here we used a semi-automatic parameterization and setup procedure, which although might not be the most accurate approach offers many advantages in screening campaigns of for instance putative drugs. Thus, the current study offers some insight how well such an approach can predict essential quantities.

In a previous benchmark of the MARTINI CG model, considerable errors were observed for the estimates of solvation free energies, whereas estimates of the partition coefficients were excellent [7]. Therefore, one of the aims of this study was to investigate if the results could be improved by using a hybrid AA/CG model. From the results presented herein, the answer is unfortunately ‘No’. The estimates of solvation free energies in hexane, octanol and the non-polarizable water model differ very little when comparing the usage of CG or AA/CG solutes (see Table 2; Fig. 2). Using all three models, we obtain mean absolute deviations larger than 10 kJ/mol and slopes close to zero. This shows that there is some underlying physics that is missing in the MARTINI model, which is not entirely surprising considering the simplicity of the model. The only slight improvement is seen when using a polarizable water model, where especially the estimates of polar compounds are improved (see Table 4), although the MAD is still larger than 10 kJ/mol. This improvement can be traced to the direct electrostatic interaction between the CG solvent and the AA solute model, making both all solutes distinguishable and improving upon the description of electrostatics that is especially important for polar solutes. However, as shown by the BEDROC analysis (see Table 4), the poorest estimates are observed for cyclic compounds. Here a re-parameterization is probably necessary, although it is unclear whether the improvement should be made on the automatic small-molecule parameterization procedure or on the MARTINI bead model. When designing the automatic parameterization procedure there were some unresolved issues on how to best weight the individual atoms in cyclic compounds, which affects the selection of bead types [7] and thus it seems that this weighting scheme is worth investigating further. Furthermore, it would be advantageous to work on a direct coupling of the van der Waals interactions, to improve the estimates of solutes in hexane and octanol. As it is now, only the water model is polarizable and thus directly coupled to the AA solute model.

Another aim of this work was to compare the MARTINI AA/CG model to the ELBA AA/CG model that previously has been extensively benchmarked on solvation free energies and partition coefficients [21, 22]. On this point, we find that the ELBA AA/CG model clearly outperforms the MARTINI AA/CG model on the estimation of solvation free energies; for instance, the MAD is at most 4 kJ/mol when predicting solvation free energies with the ELBA model. However, both models perform equally well on partition coefficients with a MAD less than one log units and with more than 80% of the estimates having the correct sign. This shows that there is a considerable degree of error cancellation with the MARTINI model that provides excellent results in this situation, but perhaps not in others. Therefore, it seems that the MARTINI AA/CG model still could have a potential usage, although improvements are necessary and probably possible. On the technical side, the MARTINI AA/CG model could be improved by implementing a multiple timestep integrator, separating the CG–CG forces and the other forces, allowing a larger timestep and more efficient simulations. Such an implementation was accomplished for the ELBA AA/CG model with good performance as a result [20]. To summarize, we conclude that the hybrid MARTINI model could become useful in simulating interesting (bio)chemical phenomena, but improvements to efficiency, direct coupling between the CG and AA levels and re-parameterization of cyclic compounds are necessary.

## Electronic supplementary material

Below is the link to the electronic supplementary material.

Supplementary material 1 (XLSX 93 KB)
